# Shocks and health care in Latin America and the Caribbean

**DOI:** 10.3389/fpubh.2025.1604424

**Published:** 2025-07-14

**Authors:** Marcia C. Castro, Jamie Ponmattam, Emily A. FitzGerald

**Affiliations:** Department of Global Health and Population, Harvard T.H. Chan School of Public Health, Boston, MA, United States

**Keywords:** disaster, shock, healthcare, health system resilience, Latin America and the Caribbean

## Abstract

**Background:**

The Latin American and the Caribbean (LAC) is one of the most disaster-prone regions worldwide, and the frequency and intensity of disasters is expected to increase. We propose typologies of shocks considering healthcare resilience to examine how the risk of shocks varies across LAC and how previous shocks and their impacts in LAC fit into these categories.

**Methods:**

We classify shocks into natural, anthropogenic and climate-related, and build on the literature to develop a 2×2 classification considering health care resilience and trust in government. Using the INFORM risk we categorize countries into risk groups considering indicators of governance and access to healthcare as proxies for trust in government and health care resilience, respectively. We discuss the 2×2 classification considering examples of health impacts of shocks, highlighting strengths and weaknesses of national responses, and use excess death ratios during the COVID-19 pandemic to demonstrate how health impacts correspond to the 2×2 typology.

**Results:**

Based on the available literature, the proposed 2×2 classification reflects the recent consequences of shocks in LAC countries. Overall, areas where healthcare access and trust in government were weak had the most devastating impacts. However, strong access to healthcare is not a sufficient condition determining the impact of a shock, as evidenced during the COVID-19 pandemic. For the most part, countries lack a detailed shock management plan.

**Discussion:**

Countries in the LAC region have historically been unprepared to manage shocks. In the absence of a comprehensive and multisectoral shock management plan, countries will continue to act in a reactive way, after a shock, as most of the examples discussed in our analysis illustrate. A shock management plan is an important step to build resilient health systems.

## Introduction

Natural, anthropogenic, and climate-related shocks affect societies in innumerable ways (1). Regarding health, such shocks have been shown to increase mortality and morbidity due to several causes, including infectious and non-communicable diseases, malnutrition, injuries, mental health, and respiratory illness. In addition, they can also trigger population displacement, which further exacerbates the challenges faced by affected populations (2–4). While the effects of shocks on health outcomes are well documented, they also have the potential to hinder the functionality of health systems, thereby exacerbating the adverse health outcomes for the affected populations in the short and long term (5, 6).

Shocks may affect health care through a combination of physical destruction of infrastructure (e.g., buildings, equipment, connectivity), workforce shortages, high levels of stress and burnout among health workers [particularly the primary health care workforce, who is often the first line of healthcare workers for populations in the aftermath of shocks (7)], disruption of health services, reduced access to health care (including complete isolation of some communities and/or areas), over capacity, decreased quality of services, and financial burden (6, 8, 9). In addition, population displacement may alter the demand for services and the exposure to pathogens in receiving areas. Specifically, in-migrants may be exposed to new pathogens not existing in the sending areas or may carry pathogens that were not previously circulating in receiving areas.

The magnitude of these effects depends on several factors, including the type, onset and duration of shocks, the local demographics (older vs. younger populations), the pattern of local inequalities (e.g., precarious housing and access to infrastructure due to fast and unplanned urban growth), and type and coverage of health care available to the population. Most importantly, it depends on the extent to which affected areas are prepared to respond to these shocks. For health care, specifically, resilient health systems are able to prevent, prepare for, detect, adapt to, respond to, and recover from shocks, without disruption of essential services and without compromising the quality of those services (10, 11). The Latin American and Caribbean (LAC) region is the second most disaster-prone region on a global scale; between 2000 and 2022, more than 190 million people were affected by 1,534 disasters in LAC (12). In 2023 alone, 1.3 million people were exposed to severe drought in just eight countries (Bolivia, Colombia, El Salvador, Guatemala, Honduras, Nicaragua, Peru and Venezuela) due to the 2023–2024 El Niño event, and the Amazon region in Brazil witnessed the most severe drought on record. Beyond natural disasters and events, environmental accidents and changes and political instability and violence have affected many countries in the region.

Here we characterize shocks by type, and propose a conceptual framework to classify shock impacts considering health care resilience. We examine how the risk of shocks vary across LAC, and how healthcare and governance indicators support this conceptual framework. Lastly, we discuss examples of how previous shocks in LAC fit into this conceptual framework.

## Types and typologies of shocks

**Figure 1:**
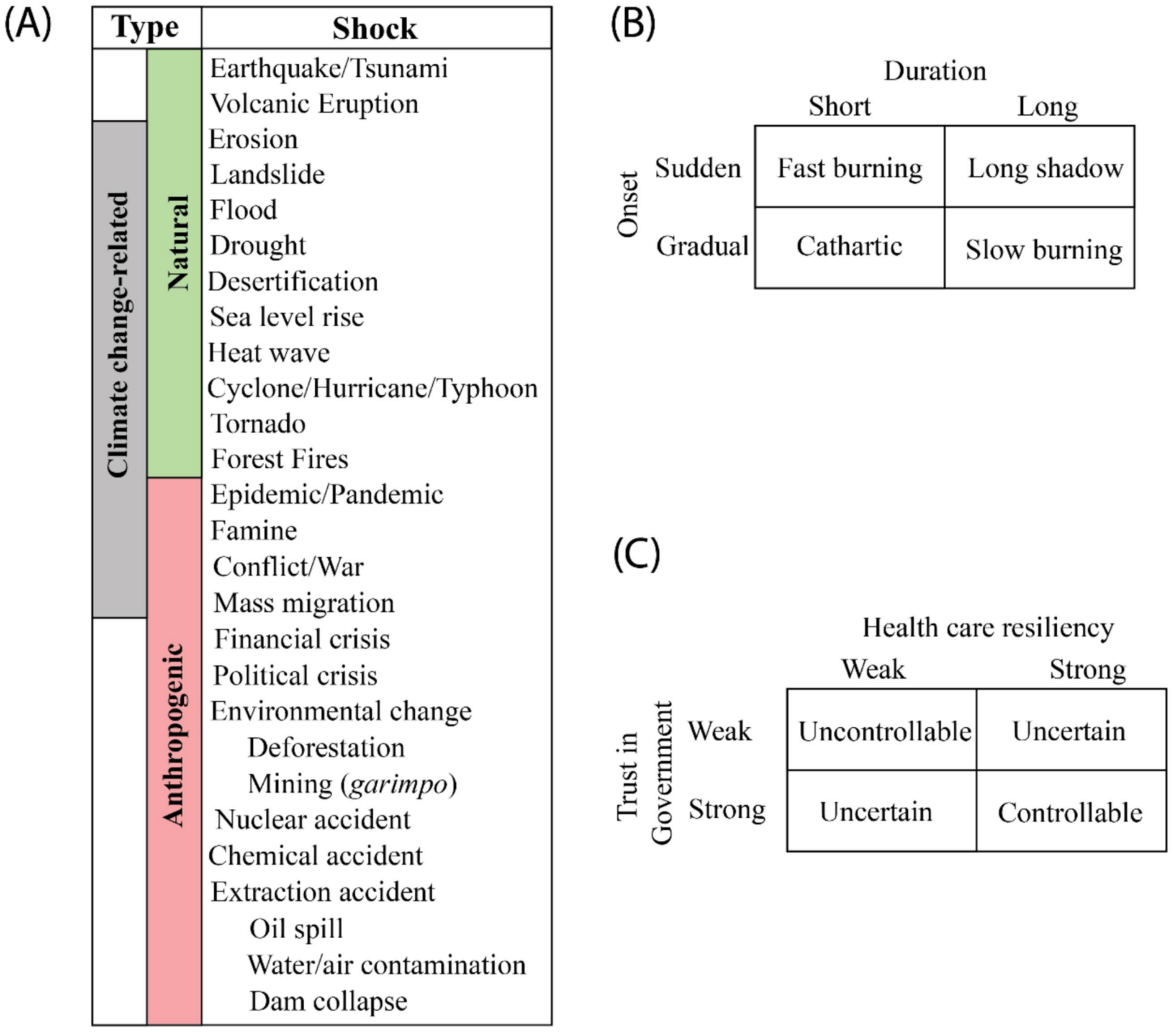
Type and categories of shocks. **(A)** Shocks classified by natural or anthropogenic causes and by possible relationships with climate change. Categories of shocks considering their onset and duration **(B)**, local trust in government and health care resilience **(C)**, and predictability and health care resilience.

While the terms “shock” and “disaster” could be considered interchangeable, “disaster” tends to evoke natural events, many related to climate. Although the definition of disaster (13–15) technically includes non-natural events, much of the focus of the disaster-related literature focuses on natural disaster risk management and prevention (15), and not necessarily on preventing political or economic shocks, or environmental accidents that can disrupt societies and communities. Here, we use “shocks” as a broader category that also includes crises/events that disrupt society but are not natural events (e.g., political crisis). We classify shocks into three non-mutually exclusive categories: (1) caused by nature (natural), (2) caused by human activity (anthropogenic), and (3) climate-related, which overlaps with the previous two types (Figure 1A). Natural disasters have had the most attention in the literature. Anthropogenic shocks include shocks from industry activity (e.g., chemical, nuclear, and extraction accidents), exploitative and illegal land use patterns (e.g., deforestation and mining), wars, political and financial crises, famine, and public health emergencies (e.g., epidemics and pandemics). Climate-related shocks are often natural disasters which have increased in both frequency and intensity recently due to changes in climate resulting from greenhouse gas emissions (16, 17),; some of the anthropogenic disasters may also be climate related.

Shocks do not necessarily occur in isolation. Some anthropogenic shocks may result from other types of shocks. For example, famine could result from droughts that affect agricultural production, conflicts/wars, and mass migration, among other reasons. The emergence or re-emergence of pathogens may be triggered, for example, by deforestation, temperature change, and practices of wildlife hunting. In both cases, however, effective surveillance and weather monitoring, coupled with mitigation and fast response strategies are critical to minimize the impacts of those shocks. Considering the onset and subsequent development of the shock over time (duration), ‘t Hart and Boin (18) proposed a widely used 2×2 classification (Figure 1B). A fast-burning crisis happens when a shock has a sudden start and ends quicky. Examples could be a forest fire that ends quickly, and a laboratory accident with a pathogen that is promptly contained with no spillovers outside the facility. When the shock is sudden but develops over a long period, and thus takes time to end, then a long shadow crisis emerges. Shocks that end quickly after a gradual onset are classified as cathartic crisis. Wars and conflicts are examples of such crises, although the consequences persist after the end, as they could have triggered other shocks (e.g., famine). The final classification is the slow burning (or creeping) crisis, when the onset is gradual, and it has a long duration. The climate crisis is an example of this category, as are three climate-related shocks included in Figure 1A that develop over a long time and are likely to be irreversible, namely sea level rise, erosion, and desertification. In such cases, while the crisis does not end per se, the longer the implementation of actions to mitigate their impact, the worse the societal and environmental consequences.

While ‘t Hart and Boin considers the characteristics of the shock/crisis in the classification of its impact, we build on this literature to consider how the context and health systems of the country in which the shock occurs affects its outcome. We are specifically interested in exploring how healthcare resilience and trust in governance impacts shock aftermath (19, 20). We chose to address trust as it plays an important role during crises, as exemplified by the Covid-19 pandemic (21, 22). In this conceptualization, three categories of crises may unfold (Figure 1C). When both health care resilience and trust in government are weak it is likely that an uncontrollable shock will emerge, with potentially high losses for society. When both are strong, it is expected that the shock can be mitigated and result in minimum losses. However, when one is weak and the other strong, it is unclear whether resilience can mitigate lack of trust or trust can mitigate the lack of resilience. In this case, how the shock will unfold in unclear.

The 2×2 classification is a simplification of reality because many factors determine the outcome of shocks, and because shocks may occur concurrently (e.g., flooding and landslide; political crises and mass migration) or as a chain of events (e.g., earthquake followed by a tsunami and flooding). In addition, some shocks may be completely unexpected (cases when there was no historical record of such a shock having happened before in the area) and therefore the consequences could be potentially catastrophic, regardless of how strong health care resilience and government trust are. Nevertheless, these classifications may facilitate the examination of shocks, their consequences, and potential responses (23).

## Risk of shocks in Latin America and the Caribbean

Risk is conceptualized by the Intergovernmental Panel on Climate Change (IPCC) as an interaction between hazard (a possible event that may have adverse effects on a population—e.g., an earthquake), exposure (the elements, like population or infrastructure, in an area where the hazard may occur), and vulnerability (the likelihood of the exposed elements suffering adverse effects from the hazard event) (24). Therefore, the risk of a given hazard depends on how exposed an element is and how vulnerable the element is to the hazard. Given the high burden of shock-related impacts in LAC and to better understand the challenges that LAC countries face, we sought to characterize countries within the region based on their risk levels and to relate health care and governance proxies to the above typology (Figure 1C).

The INFORM Risk index is the most comprehensive global risk assessment, identifying countries at risk of both humanitarian and natural crises (25). INFORM is a composite indicator comprised of three dimensions of risk: (1) hazard and exposure, (2) vulnerability, (3) lack of coping capacity (26). Unlike the IPCC, INFORM combines hazard and exposure into one dimension, while disaggregating vulnerability into susceptibility to hazard, and lack of coping capacity. The INFORM risk score ranges from zero (lowest risk) to 10 (highest risk) and is calculated as $\begin{array}{l} \text{Risk}=\text{Hazard}\&\text{Exposur}e^{1/3}\times \text{Vulnerabilit}y^{1/3}\times \text{Lack of Coping}\;\;\;\\ \text{Capacit}y^{1/3} \end{array}$

Each of these dimensions is comprised of category indices which are themselves composed of component indices. The hazard and exposure dimension is divided into natural (earthquake, tsunami, river flood, coastal flood, tropical cyclone, drought, and epidemics), and human (conflict intensity and projected conflict probability) indices. The vulnerability dimension has two indices: socio-economic (development and deprivation, inequality, and aid dependency), and vulnerable groups (uprooted people, and other vulnerable groups). Lastly, the lack of coping capacity dimension is divided into institutional (disaster risk reduction, and governance), and infrastructure indices (communication, physical infrastructure, and access to health care and education). Further details about indicators that comprise these indices can be found in the INFORM technical documentation (25).

To characterize risk levels in this regional analysis, we categorized LAC countries considering the quintiles of each INFORM dimension. We compared the country’s risk categorization with dimension categorization and assessed changes in categories over time.

To quantify the typology of shock (Figure 1C) and relate it to the INFORM risk, we considered the two component indices in the lack of coping capacity dimension, namely governance and access to health care (Supplementary Table 1), as proxies for trust in government and health system resilience, respectively. We selected these as proxies because they correlate well with other validated indices for trust in governance, perceptions of corruption in government, and healthcare accessibility (Supplementary Table 2S7). For each index, we categorized it as below or above the median value, with higher values associated with lower access to healthcare and governance scores. Low access to healthcare and governance categories serves as proxies for weak health care resiliency and trust in government categories. We conducted a sensitivity analysis using other validated indices (Supplementary Figure 1).

**Figure 2:**
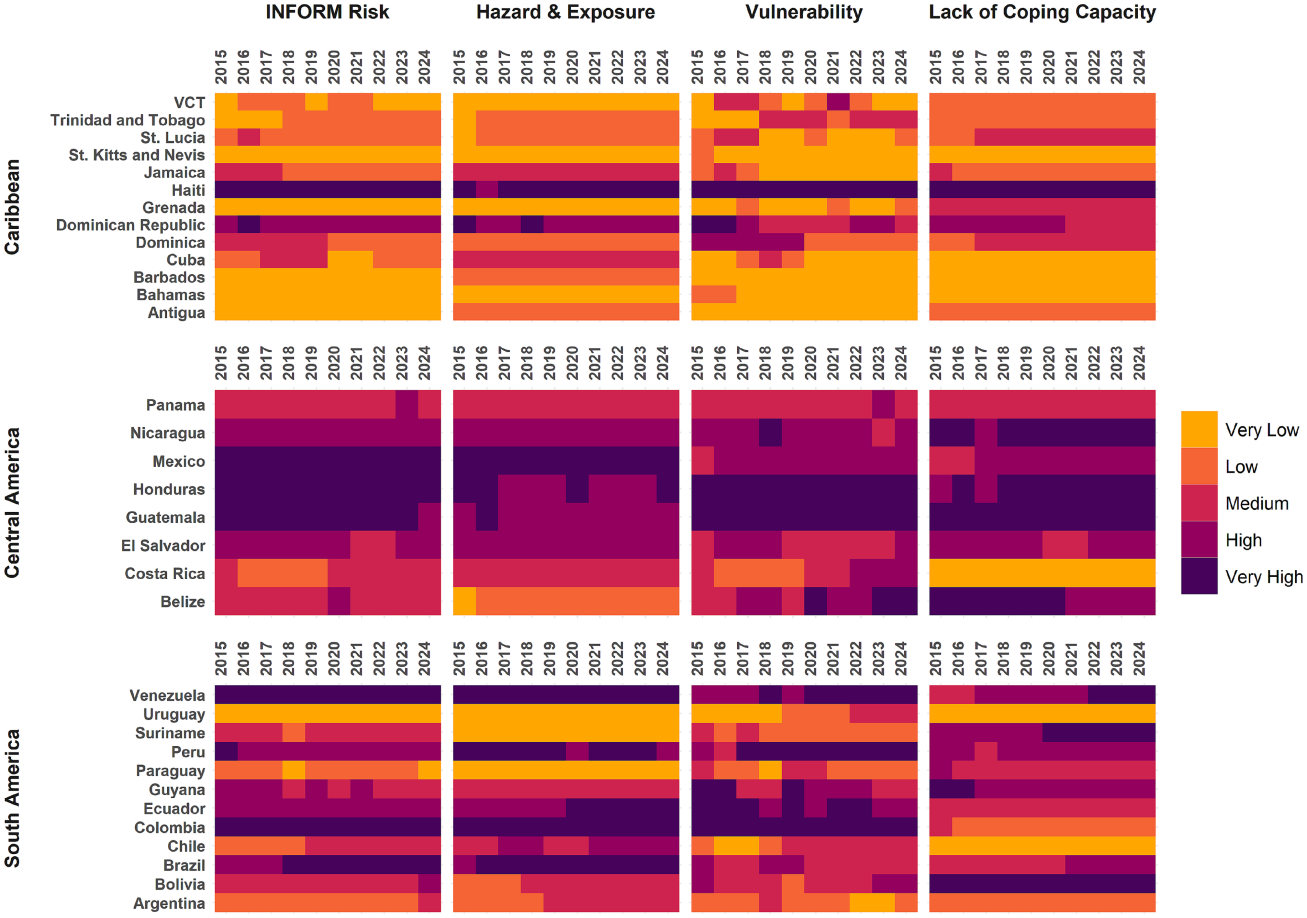
INFORM risk index for LAC countries, 2015–2024. VCT = Saint Vincent and the Grenadines.

Based on INFORM, Haiti has the highest risk index in 2024, 7.2 (Table 1) and has consistently scored highest over time in all dimensions (Figure 2), except for the hazard and exposure dimension. Five other countries had a risk index above 5, namely Colombia, Honduras, and Venezuela (risk index of 5.6), Mexico (risk index of 5.5), and Brazil (risk index of 5.2). Except for Haiti and the Dominican Republic, countries in the Caribbean (those with blue font in Table 1) do not have high scores in any dimension of INFORM. Conversely, no country in South America has an overall risk index that could be considered low or very low.

**Table 1:** INFORM Risk Index for Latin America and the Caribbean in 2024 detailed by its three dimensions (Hazards & Exposure, Vulnerability and Lack of Coping Capacity).

		INFORM Risk Index				
		Very Low [1.8–2.5]	Low (2.5, 2.98]	Medium (2.98, 3.72]	High (3.72, 4.9]	Very High (4.9,7.2]
Hazard & Exposure	**Very Low** [1.3, 1.8]	Paraguay, Uruguay Bahamas, Grenada, Saint Kitts and Nevis, St. Vincent & Grenadines		Suriname		
	**Low** (1.8, 2.4]	Antigua and Barbuda, Barbados	Dominica, Saint Lucia, Trinidad and Tobago	Belize		
	**Medium** (2.4, 3.1]		Cuba, Jamaica	Argentina, Guyana Costa Rica, Panama	Bolivia	
	**High** (3.1,5.3]			Chile	Peru Dominican Republic El Salvador, Guatemala, Nicaragua	
	Very High (5.3, 8.8]				Ecuador	Brazil, Colombia, Venezuela Haiti Honduras, Mexico
Vulnerability	**Very Low** [1.1, 2.4]	Antigua and Barbuda, Bahamas, Barbados, Saint Kitts and Nevis, St. Vincent & Grenadines	Cuba, Jamaica			
	**Low** (2.4, 3.2]	Paraguay Grenada	Dominica, Saint Lucia	Argentina, Suriname		
	**Medium** (3.2, 4.0]	Uruguay	Trinidad and Tobago	Chile, Guyana Panama	Dominican Republic	Brazil
	**High** (4.0, 4.9]			Costa Rica	Bolivia, Ecuador El Salvador, Nicaragua	Mexico
	Very High (4.9, 6.7]			Belize	Guatemala Peru	Colombia, Venezuela Haiti Honduras
Lack of Coping Capacity	**Very Low** [2.5, 3.2]	Uruguay Bahamas, Barbados, Saint Kitts and Nevis	Cuba	Chile Costa Rica		
	**Low** (3.2, 3.8]	Antigua and Barbuda, St. Vincent & Grenadines	Jamaica, Trinidad and Tobago	Argentina		Colombia
	**Medium** (3.84 4.1]	Paraguay Grenada	Dominica, Saint Lucia	Panama	Ecuador Dominican Republic	
	**High** (4.1, 4.96]			Guyana Belize	Peru El Salvador	Mexico Brazil
	Very High (4.96, 7.2]			Suriname	Bolivia Guatemala, Nicaragua	Venezuela Haiti Honduras

Categories of risk based on quintiles. The font color indicates the sub-region of LAC: green = South America, blue = Caribbean, and brown = Central America.

However, it is important to analyze the dimensions that comprise INFORM risk scores. Countries often scored differently in the three dimensions compared to their overall INFORM risk score. Mexico has the highest score in the hazard and vulnerability dimension, 8.8, followed by Brazil, 8.2, while Venezuela and Haiti scored highest in the vulnerability dimension. Similarly, although both Chile and Suriname had a risk index of 3.1, Chile scored high in the hazard and exposure dimension, while the main weakness in Suriname was the lack of coping capacity.

Countries with low or very low INFORM risk index tend to score stronger on governance and access to healthcare (Table 1). In contrast countries with very high and high INFORM risk index scores often have weak governance and access to healthcare. Some notable exceptions are Colombia, Brazil, and Mexico—while they all score high on the INFORM risk index, they have relatively strong access to healthcare.

Those categorizations offer insights on how countries may be prepared to handle shocks based. However, not every shock in countries with weak governance and weak access to health care will be uncontrollable because, as we highlighted before, many other contextual characteristics can exacerbate the impacts or act as protective factors against the shock. In addition, countries have regional and local inequalities that result in heterogeneity in subnational risk. Nevertheless, countries such as Haiti and Venezuela, both classified as having weak governance and access to health care, have struggled managing shocks over the past decade or so, as will be detailed next.

## Impacts of shocks on health care

As a disaster-prone region, LAC has historically witnessed severe disruptions in health care following shocks. The impact of these shocks depends not only on the type, intensity, duration, and predictability of the shock, but also on local coping capacity, including governance and health system resilience. Next, we highlight a few examples that illustrate the varied impacts following shocks, considering the typology proposed in Figure 1B and Table 2.

**Table 2:** LAC-INFORM governance and access to health care indices, 2024.

		Access to health care			
		Weak [3.8–7.9]	LAC-INFORM	Strong [0.1–3.7]	LAC-INFORM
governance	Weak [5.8–8.9]	El Salvador	High	Mexico	Very High
		Guatemala	High	Panama	Medium
		Honduras	Very High	Argentina	Medium
		Nicaragua	High	Brazil	Very High
		Bolivia	High	Guyana	Medium
		Ecuador	High	Paraguay	Very Low
		Peru	High	Cuba	Low
		Suriname	Medium		
		Venezuela	Very High		
		Dominican Republic	High		
		Haiti	Very High		
	Strong [3.0–5.7]	Belize	Medium	Costa Rica	Medium
		Dominica	Low	Colombia	Very High
		Grenada	Very Low	Chile	Medium
		Jamaica	Low	Uruguay	Very Low
		St. Lucia	Low	Antigua and Barbados	Very Low
		St. Vincent and Grenadines	Very Low	Bahamas	Very Low
				Barbados	Very Low
				Saint Kitts and Nevis	Very Low
				Trinidad and Tobago	Low

The font color indicates the sub-region of LAC: green = South America, blue = Caribbean, and brown = Central America. The LAC-INFORM column shows the overall risk index category for each country, as shown in Table 1. Both indicators are categorized as weak (above the median) and strong (below the median).

We expect that the impacts of shocks to be uncontrollable in areas where both the trust in government and health system resiliency are weak (Figure 1B). In LAC, countries with weak governance and weak access to health care have historically experienced and demonstrated severe impacts following shocks. The 2001 earthquake in El Salvador (magnitude 6.6) damaged 21% of health units and 7% of health centers (27). A total of 1,150 deaths were reported, over 8,000 people were injured, and 1.5 million people were affected (28). In rural Bolivia, recurrent droughts motivated people to leave farms to work as day laborers, miners, or as construction/factory workers. Aid from the government during these events was often inadequate or immaterial, leaving the population feeling distanced and “forgotten” by the government (29).

Shocks in these countries often exacerbate and worsen pre-existing inequities. For example, the deadliest and most damaging earthquake in LAC was the 2010 earthquake in Haiti (magnitude 7), with an estimated 222,570 deaths, 300,000 injured (30), 1.5 million internally displaced (31), and more than 50 hospitals and health centers damaged (32). There was extensive physical destruction of health infrastructure, disruption of services, shortage of workforce and lack of comprehensive information management systems, hindering coordination and response (Box 1). Since the 2010 earthquake, governance in Haiti has grown increasingly fragile with unchecked gang activity and increasing political violence. In the midst of dealing with this political crisis, another earthquake hit Haiti (magnitude 7.2) in 2021, resulting in further damage (33). The political crisis itself has also disrupted local health systems, with 40% of medical staff lost by the end of 2023, and 73% of hospitals in the Ouest department either ceasing operations completely or providing limited services. Many health facilities struggle to remain open due to loss of workforce and rising costs of fuel and supplies (33, 34).

BOX 1 **2010 Earthquake in Haiti.**

On January 12, 2010, Haiti was hit by a magnitude 7 earthquake near Leogane, Oest department, and experienced aftershocks of magnitude 4.5 or more for an additional 12 days. The severity of the earthquake, distribution of aftershocks, and fragile context of the country caused widespread damage. It is estimated that 222,500 people died, 300,000 were injured (30), and 1.5 million were internally displaced (31). The damage to homes and infrastructure was staggering with over 300,000 homes, 1,300 educational institutions damaged or destroyed, and over 50 hospitals and health centers collapsed or unusable (35). Many government and public administration buildings were damaged or destroyed, crippling the ability of a fragile government to lead the recovery response. Damage and losses from the earthquake totaled $7.8 billion, with 6% ($470 million) from the health sector alone (32). Immediately after the disaster, Haiti received assistance from PAHO, the World Health Organization (WHO), neighboring countries, and relief agencies to aid in healthcare and post-disaster recover (36).

Although the earthquake itself was severe, it exacerbated existing inequities and problems. As one of the poorest countries in the Western hemisphere, Haiti has a fragile health system that lack resilience to cope with and recover from a disaster (25). Even prior to the earthquake, Haiti had the lowest life expectancy in the region and an under-5 mortality rate double that of the LAC average. Almost 95% of deaths were not registered and measles immunization rate was only 58% as of 2008. About 75% of healthcare in the country is delivered by NGOs and other foreign medical providers, and nearly half of the population lacks access to basic health services, and what was offered was of subpar quality (32). Beyond the health system, Haiti has undergone near annual political, natural, and epidemic shocks since 2000. The close spacing of these events has given Haiti little time to recover and recuperate and each shock has consequences that interplay to affect the functioning of the health system (33).

To address the increased and widespread healthcare needs post-earthquake, the Ministry of Health established mobile clinics to provide primary healthcare to displaced populations or to substitute for destroyed facilities. However, in March 2010 only 72 out of 206 settlements had local access to health care including the mobile clinics (32). Additionally, only 10% of the clinics offered the minimum package of general consultation, prenatal care, pediatric consultation, neonatal care, family planning, and vaccination. Less than half offered immunizations and family planning services. Nevertheless, the quality and proximity of the mobile primary care clinic was considered superior to what was available prior to the earthquake.

In October 2010, a cholera epidemic broke out in Haiti, likely introduced by Nepalese UN peacekeepers who were a part the long-standing UN stabilization Mission in Haiti (MINUSTAH) (37). Although the cholera epidemic was not related to the earthquake, the lack of adequate WASH infrastructure pre-earthquake and the slow rebuilding post-earthquake allowed the cholera bacterium to flourish and transmit widely, leading to the first cholera epidemic in Haiti in over a century. Reconstruction efforts were slow, halting, and subject to poor project management and development.

Considering the typologies in Figure 1B, the management of this crisis was uncontrollable and catastrophic. Haiti’s health system and PHC resilience was weak, trust in the government has been low for decades, and the magnitude of the earthquake was unpredictable. While the aspirations of the Ministry of Health response were high, it was not enough to overcome a long and persistently weak healthcare system.

Following the 2017 Hurricane Maria, Puerto Rico faced a complete collapse of the health system (38). The impacts of the hurricane were exacerbated by a fragile electrical grid and pre-hurricane health system, as well as poor disaster governance (39). In addition to physical destruction of the healthcare buildings, health service provision was disrupted, and services were over capacity. Health and surveillance information were not collected which made care coordination difficult (38). Health care workers in Puerto Rico felt abandoned by the government as they were the first to feel government failures and linked the government’s actions to a longer history of ineptitude (38).

In Venezuela the political and economic crisis has led to a practical collapse of the medical system, despite the strengthening reforms of the early 2000s. Costs of medical supplies were hyperinflated, resulting in shortages of critical medications and medical equipment. Health facilities were underfunded and understaffed, with many medical professionals fleeing the country (40). In fact, according to the UN Refugee Agency (UNHCR), about 20% of the population fled the country (as of April 2024), and most remain as refugees or are internationally displaced in countries in South America. Spillover of diseases followed the mass migration (41), with neighboring regions experiencing increases in measles, malaria, vaccine-preventable diseases, and STDs (42–44). Many childhood vaccination rates fell below herd immunity rates, and maternal/infant mortality rates rose about 66% between 2014 and 2016 (45).

Still, countries can learn from previous disasters and improve the resilience of their health systems, even if they would fall under the “uncontrollable” category in our typology. In 1998, Hurricane Mitch devasted Nicaragua, destroying over 500 health centers (46). By the time of the 2016 Hurricane Otto, the Ministry of Health had improved their adaptive capacity by working with Pan American Health Organization (PAHO) and pre-positioning eight medical teams to organize and ensure sanitary conditions, and to set up necessary medical services in the aftermath of the shock (47).

When both health care resilience and trust in government are strong, we expect that the impacts of shocks can be better controlled (Figure 1B and Table 2). Costa Rica, for example, stands out as a successful example in managing the Covid-19 pandemic. The unified Digital Health Record (EDUS) enabled near-real time monitoring of patients, resources and workforce across the entire health system; essential health services were sustained; and the strong trust in the government and health care resilience were pivotal in mitigating the impacts of the pandemic (Box 2) (48).

BOX 2 **Costa Rica’s COVID-19 response.**

Costa Rica had one of the most effective responses to the Covid-19 pandemic in the LAC region. Beginning with shutdowns, the response quickly expanded to a multisectoral response (49). The government supported public health efforts by introducing economic relief measures to protect workers and ensuring access to supplies for vulnerable populations. Other entities, like the Costa Rican Electricity Institute and the National Liquor Factory also contributed by serving as an information platform and supplying alcohol and antiseptic solutions (50). While the Costa Rican Social Security Fund (CCSS) faced challenges such as supply shortages and the Ministry of Health faced social pressure to ease restrictions (49), Costa Rica was able to sustain essential health services for the population throughout the pandemic.

The COVID-19 response relied on adapting structures and processes of both the tertiary and primary healthcare systems to increase flexibility and responsiveness and prevent hospitals from overcrowding (48). In addition to creating a specialized Covid-19 center and increasing the number of intensive care unit beds, the CCSS augmented the health care workforce and hired more than 4,000 additional primary care workers. They also created 24/7 call centers to remotely monitor Covid-19 patients and began home delivery of medications for people with chronic conditions. At the ministerial and administrative levels, resource allocation and patient transfer were coordinated to prevent overcrowding and supply shortages, and governance flexibility was increased through information sharing in virtual meetings and expedited decision-making due to streamlined procurement procedures.

The underlying strengths of Costa Rica’s response lay in a robust universal public healthcare system, adaptive leadership with strong messaging, and a single, national electronic health record state unified across all levels of care (EDUS). Costa Rica created its universal health system in the 1940s, and it is considered a strong and important institution by Costa Ricans (50). Within days of the first Covid-19 case, Costa Rica declared a yellow alert status to mobilize resources and activate emergency operation centers. The Ministry of Health led the communication strategy, with daily briefings that focused on technical issues, relevant updates, and how individuals could avoid the risk of getting and transmitting Covid-19 (49). With the EDUS, CCSS could monitor occupancy rates in various facilities, and manage transfers of patients and supplies to avoid overcrowding and supply shortages.

Willingness to get a Covid-19 vaccine was high and Costa Ricans primarily trusted vaccine recommendations from local health workers, the WHO, and government health officials (51, 52). Within one year of first receiving Covid-19 vaccine doses, Costa Rica had vaccinated 78% of its population (53). Although mortality during the pandemic was higher than the pre-pandemic years, most of the additional mortality was driven by Covid-19, either directly or indirectly (48, 54).

Considering the typologies in Figure 1B, the Covid-19 pandemic could be classified as a slow-burning shock—cases and deaths gradually increased over time and the pandemic persisted for close to three years. However, the coordinated and unified response of the government along with a resilient and well-trusted healthcare system allowed Costa Rica to control the pandemic and prevented a health system collapse.

Although Chile is a medium risk country with strong governance and access to healthcare, poor government decisions and lack of preparation for the 2010 earthquake in Maule (magnitude 8.8) caused extensive damage and devastation. Following the earthquake, a lack of coordination in the government and a series of contradictory orders led to a delay in issuing a tsunami alert, resulting in high mortality. Locally, primary care teams had inadequate responses to mental health problems post-earthquake due to a lack of anticipation and planning (27, 67).

The ability of LAC countries with mixed governance and access to health care to properly manage disasters is mixed. For example, according to INFORM, Brazil has very high risk, weak governance, and high access to healthcare (Table 2). Despite having one of the largest universal health care systems, the country has had multiple disasters with devastating impacts. Flooding and mudslides following a tropical storm in 2011 in Rio de Janeiro resulted in almost 1,000 deaths and more than 30,000 people were displaced. The municipal government had no disaster management or preparation plans in place, the local health system was under-resourced and ill-prepared to triage and treat victims, and areas mostly affected had subpar house construction in zones with very high risk for disasters (55).

In addition, anthropogenic disasters in Brazil, such as the collapse of tailing dams, have also had devastating impacts. In 2015, the dam at the Samarco iron ore mine collapsed in Mariana. Mental health deteriorated and significant losses in quality of life were observed (35–37). The collapse also caused widespread environmental damage along the Doce River basin, where 43.7 million cubic meters of mine tailings were discharged. This was the worst environmental disaster ever recorded in Brazil. In 2019, the dam at the Córrego do Feijão iron ore mine collapsed in Brumadinho. As a result, 272 people died, entire villages were flattened resulting in mass migration into surrounding municipalities, and the demand for health care increased (49). Although unexpected, these shocks are a direct result of industry mismanagement and lack of strict regulations and enforcement that minimize risk in mining operations (50).

For countries with weak access to healthcare, strong preparation, action and coordination in the wake of a disaster can improve the resiliency of a health system to respond to immediate needs. For example, following the La Soufrière volcano eruption in 2021, St. Vincent and the Grenadines (VCT) demonstrated how government preparedness and integrated coordination with non-health actors, trust in government action, and primary care resilience can control a crisis. Prior to the eruption, the VCT government evacuated 20,000 people living in red and orange zones near the volcano. Health services in these regions were moved to safer districts. PAHO supported the government by supplying medical resources, developing public health messages, and providing an on-the-ground team. During evacuations, PAHO and the Ministry of Health coordinated and carried out a Covid-19 vaccination campaign and implemented syndromic surveillance, particularly in the evacuation centers. While the eruption unavoidably damaged the infrastructure, there were no deaths from the volcano and residents were able to return to their homes after the crisis (56).

## Covid-19 pandemic: a pan-regional analysis

Currently, there is no worldwide comprehensive database of health and infrastructure impacts of shocks listed in Figure 1A. Furthermore, there is a wide variation in the shocks themselves. For example, while hurricanes and earthquakes may strike many countries in the LAC region, their strength varies both between and within the same event. However, the Covid-19 pandemic presents an unique opportunity for analysis since the entire globe was immune-naïve to the SARS-CoV-2 virus. In this case, the local context and political decisions played a major role in the outcome of the pandemic (57).

To standardize comparisons across countries we calculated the excess deaths ratio (excess deaths divided by expected deaths) using the WHO excess death estimates (58). We calculated the correlation between the excess death ratio and the INFORM Governance and Access to Health Care indicators. We assessed whether the excess death ratio was above or below the median excess death ratio of the LAC region and considered the pandemic as “controlled” if the ratio was below the median and “uncontrolled” if above. We performed a similar analysis using non-INFORM indices (Supplementary Figure 2).

**Figure 3:**
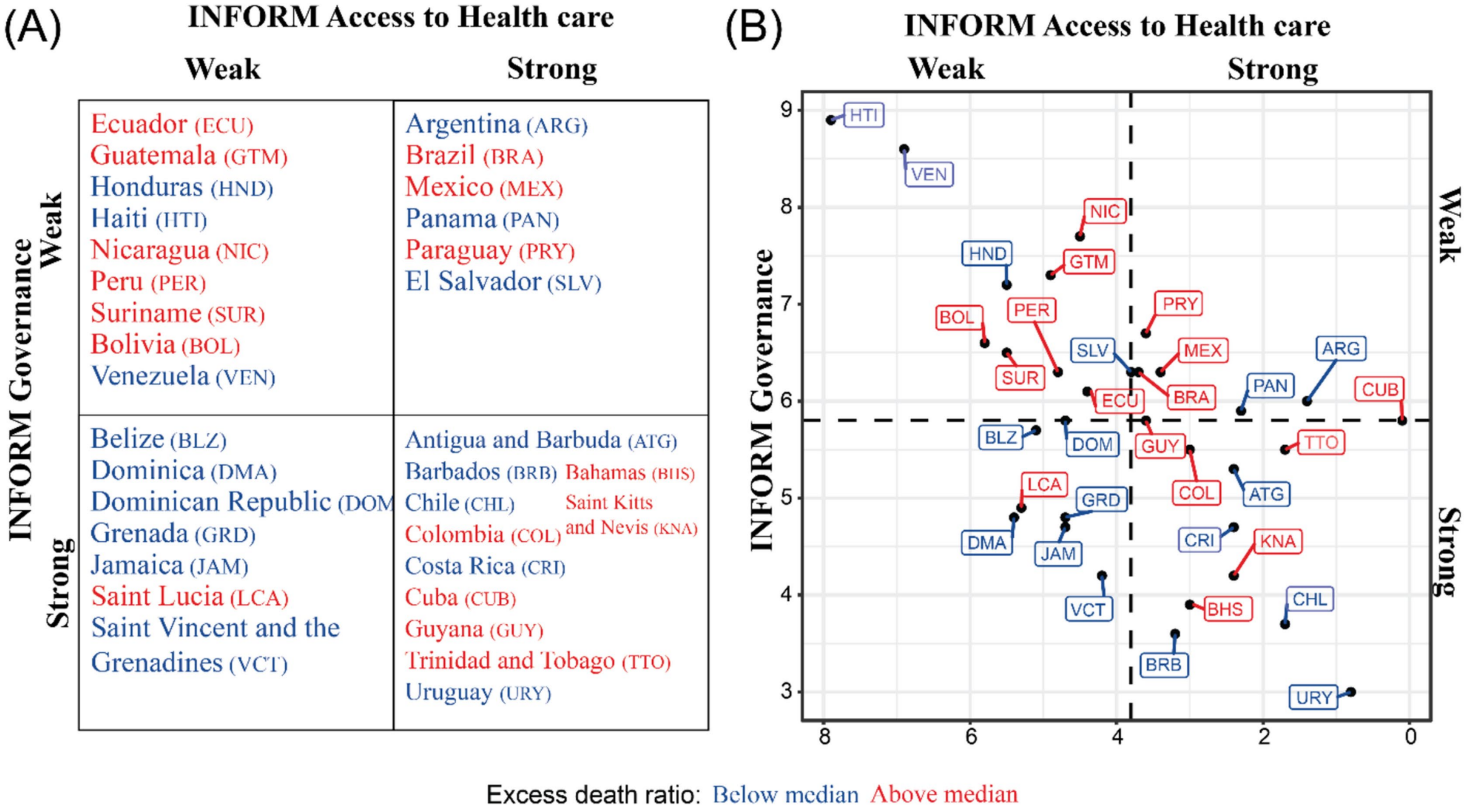
Typology based on excess Covid-19 death ratios. **(A)** 2×2 typology of countries using the INFORM index on Governance (proxy for trust in government) and Access to Health care (proxy for healthy system resilience) considering their excess Covid-19 death ratio. **(B)** Countries plotted by INFORM indices and their excess Covid-19 death ratio. INFORM indices range from 1 to 10 with 1 being the best and 10 being the worst.

Regionally, the excess deaths ratio positively correlates with the INFORM Governance indicator (0.253) while it is not correlated with the Access to Health care indicator (−0.034). However, when we compare excess death ratios with the regional median of countries across quadrants, most countries with weak access to healthcare and weak governance scores had excess death ratios above the median. Reflecting the complex dynamics of shocks, and the simplified picture of a 2×2 typology, some countries with strong governance and access to healthcare also had excess death ratios above the median (Figure 3).

These results highlight that strong governance and access to healthcare while important were not sufficient to guarantee better outcomes during the pandemic. They are supported by previous work reporting that pandemic-preparedness and health-care capacity indices/indicators were not associated with Covid-19 outcomes, but governance and trust indicators were associated with health outcomes (59). Conceptualizing the typology with excess death ratios also invites us to consider how countries within each quadrant differs in their responses to the pandemic and how that may affect their excess death ratios. Costa Rica (Box 2) was noted to have an exceptional response to the pandemic due to the actions of the government, while Colombia’s response had a mixed impact (60). Therefore, while trust in government had a strong impact during the pandemic, individual government actions and policies affected were major determinants of the pandemic outcome.

## Discussion

We presented a classification of shocks encompassing three non-exclusive categories (natural, anthropogenic and climate change related) and a typology that considered two variables: trust in government and health system resilience. Utilizing the INFORM risk index, we categorized countries within the LAC region according to their risk, detailed by three dimensions (hazard and exposure, vulnerability, and lack of coping capacity). We also used two indicators included in the lack of copying capacity dimension as proxies for trust in government and health system resilience to construct a typology of countries. Lastly, we examined how governance policies and healthcare systems have impacted various shock events across LAC, and analyzed a regional case using excess deaths during the Covid-19 pandemic.

Our results highlight four issues. First, countries in LAC are disaster prone and have historically been underprepared to manage shocks. While there have been some success cases, far more shocks have had uncontrollable impacts. The Covid-19 pandemic showed how unprepared many health systems are. Some countries are not even providing essential health services (61). Haiti and Venezuela, for example, have been devastated by decades of political and economic instability, violence, and other natural or climate-related shocks. In those cases, investing in health systems to provide reliable, affordable and accessible essential services precedes the development of disaster management plans.

Second, strong access to health care is necessary but not sufficient to mitigate the negative health outcomes of shocks. Here, it is critical that health systems are resilient and thus able to prevent, prepare for, detect, adapt to, respond to, and recover from shocks, without disruption of essential services and without compromising the quality of those services (10, 11). Most importantly, multisectoral collaborations are the core of a resilient health system and of a well-designed disaster management plan, as exemplified by Costa Rica’s response to Covid-19 (62, 63).

Third, governance and trust in government play a critical role in the unfolding of shocks. Excess death ratios during the Covid-19 pandemic were higher in countries with weaker governance and trust in government, regardless of the strength of access to healthcare. Countries that have historically had strong health care systems, such as Brazil and Mexico, had excess death ratios above the median, while countries with weaker access to health care, such as Jamaica or the Dominican Republic, had ratios less than the median. It is important to emphasize that we do not claim a causal relationship.

Finally, the progressive intensification of the frequency and severity of extreme climatic events calls for urgent disaster management plans that go beyond the health sector. Although it is estimated that the mean temperature in LAC could increase by over 2°C by 2100 (24, 64), only four countries (Brazil, Chile, Cuba, and Saint Kitts/Nevis) have developed a Health National Adaptation Plan (HNAP) separate from their national adaptation plan, and 14 have a plan under development as of 2023 (65). However, HNAPs only cover climate-related disasters and situations; other shocks such as oil spills and deforestation are not within the scope of HNAPs. Shocks from non-climatic-related events still need to be accounted for and prepared for.

From a public health perspective, a plan to manage shocks is essential to build a resilient health system (11). In the absence of such a plan, countries act in a reactive way, after the shock, as most of the examples discussed in our analysis illustrate. Management of shocks often involves four sequential phases (prevention/mitigation, preparedness, response, and recovery) (66). The prevention/mitigation phase occurs before the shock onset but needs to be revised periodically. It includes a detailed risk analysis so that risk zones can be identified, and detailed strategies to prevent the shock (if possible) or to mitigate the loss of lives and the damage to the local infrastructure. This phase requires data at a local scale that enables policy planning. Data include health, local ecology, infrastructure, inventory of previous disasters, and population detailed by age, to name a few. It is important to note that INFORM, used in our analysis, is not ideal for this phase as it is only available at the national level. The local context (e.g., biome, climate classification, vulnerability to certain types of shocks, socioeconomic characteristics, and governance) can exacerbate the impact of shocks, or act as protective factors.

The preparedness phase also takes place before the shock, but it is an ongoing process. It uses the information provided by the prevention/mitigation phase to develop detailed plans of what to do in the event of a shock and by whom, the resources needed (human, financial, equipment, and supplies), the development of communication strategies, and the implementation of early warning systems that trigger the emergence of a shock. It also involves training and rehearsal of critical situations (e.g., evacuation) to ensure a high level of readiness to respond to a shock.

The response phase happens during and immediately after the shock. It implements the plans developed in the preparedness phase, both short- and long-term (depending on the shock). It also includes assessing damage and coordinating emergency relief (local and foreign). Information on the execution of the response phase and its evaluation feeds into the first two phases to correct mistakes and continuously improve the response to future shocks.

Lastly, the recovery phase, after the disaster, includes restoring services, provision of health services whose demand increased with the shock (e.g., mental health), and reconstruction of infrastructure, among others. The duration of this phase depends on the type and intensity of shock.

Shock management plans must consider coordination between multiple sectors of the government (health, education, infrastructure, transportation, national security, etc.), community stakeholders (business, non-governmental organizations, etc.), as well as dimensions of pre-existing inequities such as gender, indigenous communities as well as the urban–rural divide. The 2017 Hurricane in Puerto Rico is just one out of many examples that demonstrate the consequences of lack of such coordination (39).

This study has limitations. It is not intended to measure causal relationships. We propose a conceptual framework to better understand how health system resilience and trust in governance can play a role in the outcomes of shocks. However, we are limited by the nature of shocks as well as the data available. The measurable and quantifiable impacts of shocks cannot be untwined from the context in which they occur, making it difficult to compare the same type of shock across different years and countries. While the Covid-19 pandemic offered a unique opportunity to compare countries experiencing the same event, other shocks such as earthquakes or hurricanes may be more impacted by the strength of the health system than trust in the government given their immediate impact. From a data availability standpoint, the INFORM index is the most comprehensive dataset to measure risk, governance and access to health care. However, it does not consider all the shocks listed in Figure 1A. Nevertheless, the shocks considered are common in LAC, and largely documented in the literature. Additionally, INFORM is only available at the national level for all countries in the LAC region. While some countries like Guatemala and Honduras have undertaken subnational risk assessments, not all countries have. Furthermore, subnational risk assessments consider different indicators in the calculation (e.g., Honduras does not include an indicator on access to health care). Although the national scale is informative for comparative analysis across the region, it is not appropriate for sub-national planning purposes, and hampers attempts to assess inequities in country response. A limitation with the proposed typology is that the health care access index is a crude proxy for resilience, but it is the best measure available. Also, for policy-planning purposes in specific countries, the typology may be too simple. Existing national disaster classification systems should be used for practical and local decision-making since they are tailored to the country’s context.

In summary, considering the vulnerability of LAC countries to shocks, the historical record of damage, and the likely scenario of more frequent and intense extreme climatic events, it is critical that countries strengthen their health systems, guaranteeing the consistent delivery of essential health services, and then implementing the necessary steps to build resilience in the health system. Failing to do so will result in damage and loss of lives that could be prevented or mitigated.
