# Supplementary material for: Shocks and health care in Latin America and the Caribbean
Source: Front Public Health. 2025 Jul 14;13:1604424. doi: 10.3389/fpubh.2025.1604424 (PMC12301396; doi:10.3389/fpubh.2025.1604424)
Supplement: Supplementary file 1 [file Data_Sheet_1.docx]

Supplementary Material

**Contents**

[**Table 1. Indicators that comprise the INFORM Governance and Access to Healthcare indices** 2](#_Toc200311499)

[**Table 2 - Correlation between INFORM scores and several indicators extracted from other sources.** 3](#_Toc200311500)

[**Fig. 1 Typology using (a) the Universal Healthcare Index (UHC) and the World Bank Control of Corruption indices and (B) INFORM indicators.** 4](#_Toc200311501)

[**Fig. 2 Typology based on excess Covid-19 death ratios**. 5](#_Toc200311502)

# **Table 1. Indicators that comprise the INFORM Governance and Access to Healthcare indices**

| **Governance** | **Access to Healthcare** |
| --- | --- |
| Government effectiveness | People using at least basic sanitation services (% of population) |
| Corruption perception index | People using at least basic drinking water services (% of population) |
| HFA (Hyogo Framework for Action) Scores | Physicians Density |
|  | Percentage of the target population with access to 3 doses of diphtheria-tetanus-pertussis (DTP3) |
|  | Percentage of the target population with access to measles-containing-vaccine second-dose (MCV2) |
|  | Percentage of the target population with access to pneumococcal conjugate 3rd dose (PCV3) |
|  | Current health expenditure per capita |
|  | Maternal Mortality Ratio (modeled estimate) |

# **Table 2 - Correlation between INFORM scores and several indicators extracted from other sources.**

| **Governance** | **Correlation** |
| --- | --- |
| World Bank Country Policy and Institutional Assessment – available at <https://databank.worldbank.org/source/country-policy-and-institutional-assessment> | -0.983* |
| Corruption Perception Index – available at <https://www.transparency.org/en/cpi/2024> | -0.962* |
| Corruption Perception Index Rank – available at <https://www.transparency.org/en/cpi/2024> | 0.957* |
| Varieties of Democracy (V-Dem) regime corruption – available at <https://www.v-dem.net/> | 0.888* |
| Varieties of Democracy (V-Dem) political corruption – available at <https://www.v-dem.net/> | 0.885* |
| Varieties of Democracy (V-Dem) executive corruption – available at <https://www.v-dem.net/> | 0.850* |
| Varieties of Democracy (V-Dem) public sector corruption – available at <https://www.v-dem.net/> | 0.747* |
| Percent that has no trust in government – available at [https://www.vanderbilt.edu/lapop/](https://www.vanderbilt.edu/lapop) | 0.805* |
| World Bank Control of Corruption - available at <https://www.worldbank.org/en/publication/worldwide-governance-indicators> | -0.947* |
| World Bank Government Effectiveness – available at <https://www.worldbank.org/en/publication/worldwide-governance-indicators> | -0.940* |
| World Bank Rule of Law – available at <https://www.worldbank.org/en/publication/worldwide-governance-indicators> | -0.890* |
| World Bank Percent rank of Control of Corruption – available at <https://www.worldbank.org/en/publication/worldwide-governance-indicators> | -0.945* |
| World Bank Percent rank of Government Effectiveness – available at <https://www.worldbank.org/en/publication/worldwide-governance-indicators> | -0.950* |
| World Bank Percent rank of Rule of Law – available at <https://www.worldbank.org/en/publication/worldwide-governance-indicators> | -0.888* |
| **Access to Healthcare** | **Correlation** |
| International Health Regulations (IHR) States Parties Self-Assessment (SPAR) – available at <https://www.who.int/emergencies/operations/international-health-regulations-monitoring-evaluation-framework/states-parties-self-assessment-annual-reporting> | -0.459* |
| Universal Health Care (UHC) Service Coverage Index – available at <https://www.who.int/data/gho/data/indicators/indicator-details/GHO/uhc-index-of-service-coverage> | -0.770* |
| Healthcare Access and Quality (HAQ) Index – available at <https://www.healthdata.org/research-analysis/library/assessing-performance-healthcare-access-and-quality-index-overall-and> | -0.807* |

*Indicates correlation is as expected.


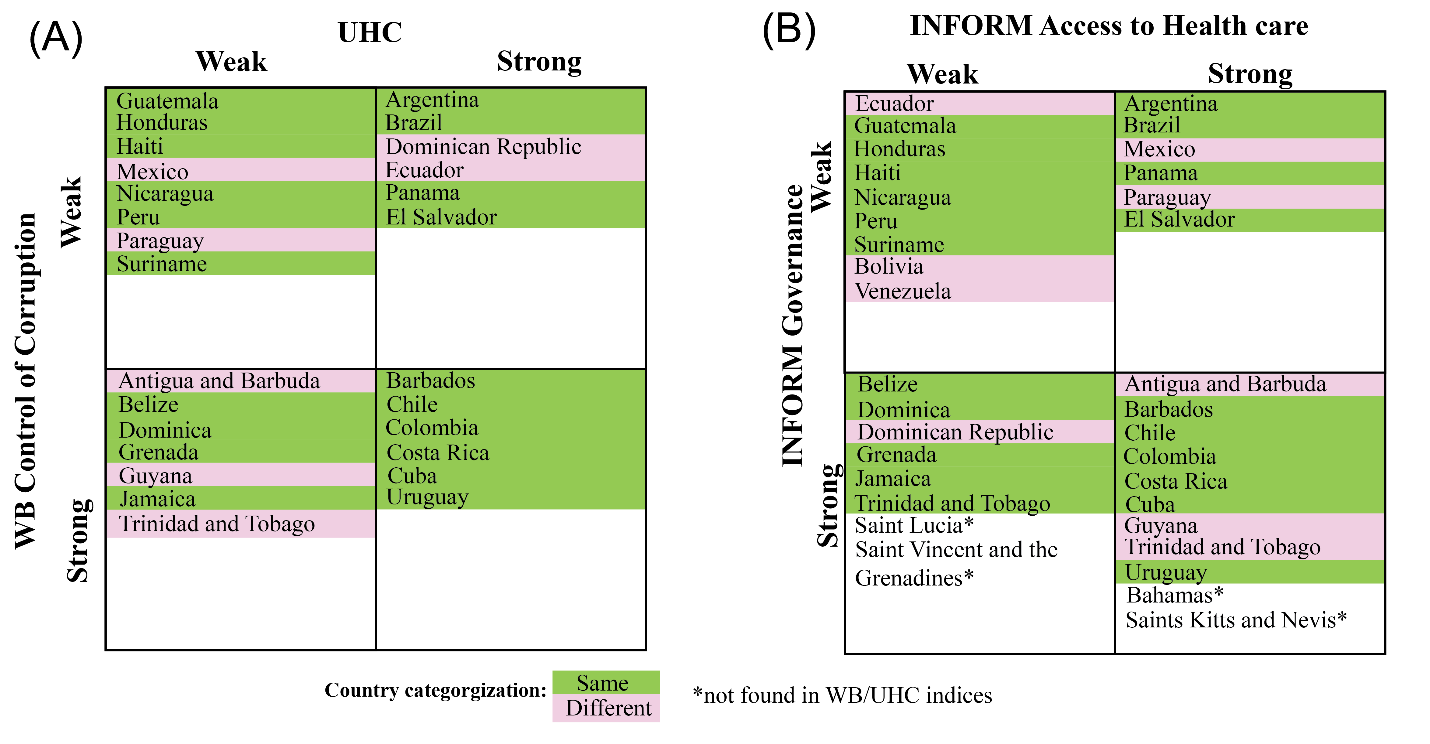


# **Fig. 1 Typology using (a) the Universal Healthcare Index (UHC) and the World Bank Control of Corruption indices and (B) INFORM indicators.**


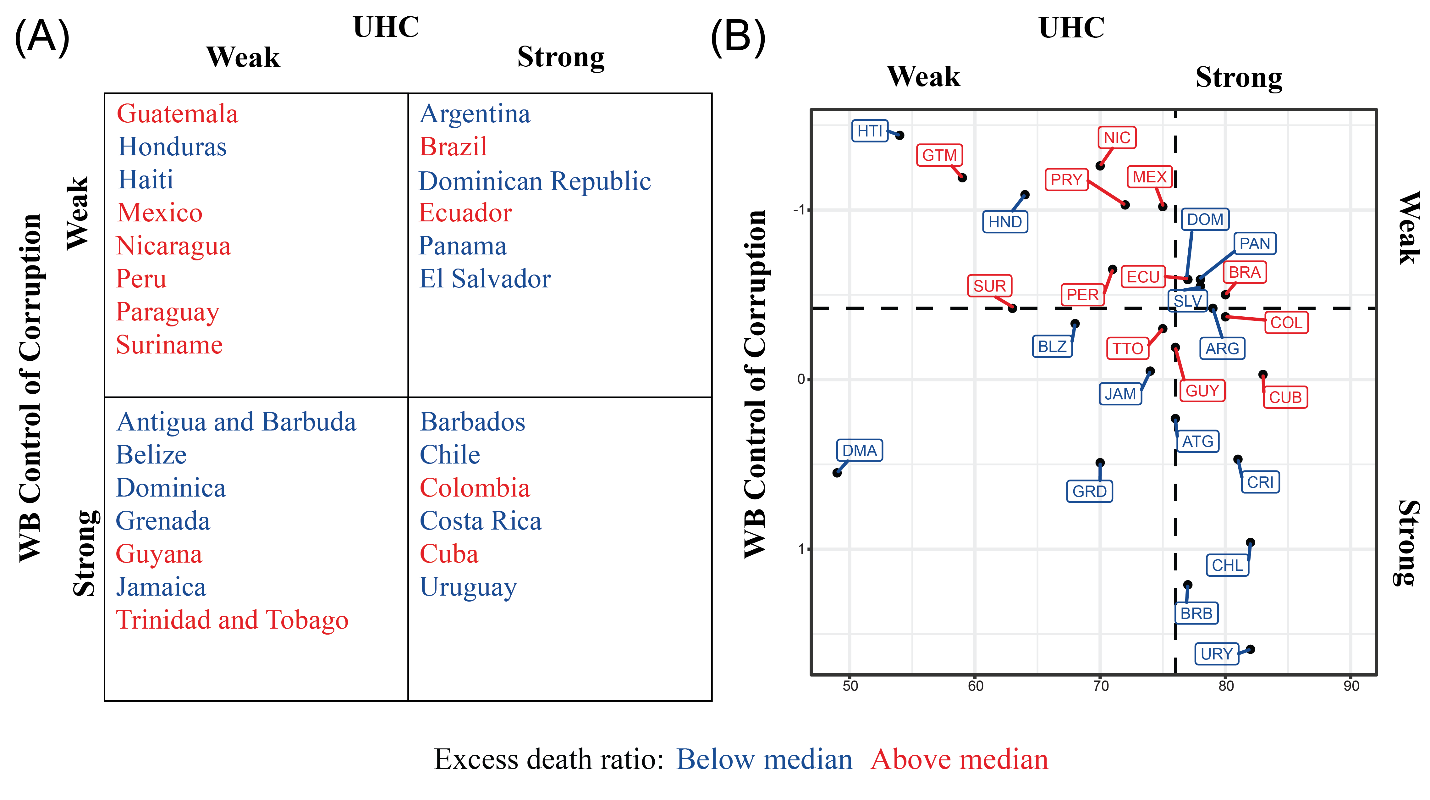


# **Fig. 2 Typology based on excess Covid-19 death ratios** (A) 2x2 typology of countries using the World Bank (WB) Control of Corruption (proxy for trust in government) and Universal Health Care index (proxy for healthy system resilience) considering their excess Covid-19 death ratio. (B) Countries plotted by World Bank (WB) Control of Corruption (proxy for trust in government) and Universal Health Care index and their excess Covid-19 death ratio.
